# Characterization and Functional Analysis of *PEBP* Family Genes in Upland Cotton (*Gossypium hirsutum* L.)

**DOI:** 10.1371/journal.pone.0161080

**Published:** 2016-08-23

**Authors:** Xiaohong Zhang, Congcong Wang, Chaoyou Pang, Hengling Wei, Hantao Wang, Meizhen Song, Shuli Fan, Shuxun Yu

**Affiliations:** State Key Laboratory of Cotton Biology, Institute of Cotton Research of CAAS, Anyang, 455000, Henan, People’s Republic of China; USDA-ARS Southern Regional Research Center, UNITED STATES

## Abstract

Upland cotton (*Gossypium hirsutum* L.) is a naturally occurring photoperiod-sensitive perennial plant species. However, sensitivity to the day length was lost during domestication. The phosphatidylethanolamine-binding protein (PEBP) gene family, of which three subclades have been identified in angiosperms, functions to promote and suppress flowering in photoperiod pathway. Recent evidence indicates that PEBP family genes play an important role in generating mobile flowering signals. We isolated homologues of the *PEBP* gene family in upland cotton and examined their regulation and function. Nine *PEBP*-like genes were cloned and phylogenetic analysis indicated the genes belonged to four subclades (*FT*, *MFT*, *TFL1* and *PEBP*). Cotton *PEBP*-like genes showed distinct expression patterns in relation to different cotton genotypes, photoperiod responsive and cultivar maturity. The *GhFT* gene expression of a semi-wild race of upland cotton were strongly induced under short day condition, whereas the *GhPEBP2* gene expression was induced under long days. We also elucidated that GhFT but not GhPEBP2 interacted with FD-like bZIP transcription factor GhFD and promote flowering under both long- and short-day conditions. The present result indicated that *GhPEBP*-like genes may perform different functions. This work corroborates the involvement of *PEBP*-like genes in photoperiod response and regulation of flowering time in different cotton genotypes, and contributes to an improved understanding of the function of *PEBP*-like genes in cotton.

## Introduction

The phosphatidylethanolamine-binding protein (PEBP) family genes are found in all three major phylogenetic divisions of bacteria, archaea, and eukaryotes [1–3]. Conserved sequence regions in PEBP proteins provide evidence for an ancient common origin of a basic protein functional unit. Thus, PEBP domains specific to bacteria and archaea have been identified. Animal PEBP proteins act as Raf kinase inhibitors [4]. PEBP-related proteins have been discovered in many plant species, including snapdragon [5], *Arabidopsis* [6], grapevine [7], lombardy poplar [8], legume [9], barley [10], tomato [11], rice [12] and maize [13]. Most plant PEBP-like proteins contain conserved eukaryotes-specific regions [14] and are resolvable into three phylogenetically distinct subclades, which comprise the FLOWERING LOCUS T (FT)-like proteins, the TERMINAL FLOWER1 (TFL1)-like proteins and the MOTHER OF FT AND TFL1 (MFT)-like proteins. *FT*/*TFL1*-like genes are mainly involved in flowering-time regulatory pathways, whereas *MFT*-like genes are involved in seed development and germination [15,16].

Plants have developed mechanisms to control the vegetative and reproductive growth by integration of environmental and developmental cues. The plant life cycle is divided into distinct developmental phases based on the morphological and functional characteristics of the organs that differentiate from the shoot apical meristem. In *Arabidopsis thaliana*, flowering is initiated by four regulatory pathways (photoperiodic, temperature, age and gibberellins) that converge on three integrator genes, namely *FT*, *SUPPRESSOR OF OVEREXPRESSION OF CONSTANS 1* (*SOC1*) and *LEAFY* (*LFY*) [17,18]. Photoperiodic pathway integrate the circadian clock and light, and are involved in the expression and regulation of genes such as *CONSTANS* (*CO*), *FLAVIN BINDING*, *KELCH REPEAT*, *F-BOX 1* (*FKF1*) and so on [19–21]. Activation of *FT* is achieved largely through regulation of the transcription factors *CO*, *TARGET OF EAT1* (*TOE1*) and other genes involved in photoperiodic pathways [22,23]. The FT protein is a major component of the florigen signal, which is transported in the phloem from leaves to the shoot apex where it promotes flowering and interacts with the bZIP transcription factor *FLOWERING LOCUS D* (*FD*) to activate floral meristem identity genes *APETALA1* (*AP1*) and *LFY* [24–27]. In Monocotyledon, rice FT homologue protein Hd3a also interacts with 14-3-3 and OsFD1 proteins to form a florigen activation complex, which induces expression of the *Arabidopsis AP1* homologue gene *OsMADS15* [28,29].

Most plant *PEBP*-related genes have been identified from mutants that show altered inflorescence architecture. In *Arabidopsis*, two *FT*-like genes, *FT* and *TWIN SISTER OF FT* (*TSF*), are floral activators whose mutants are late-flowering [18,30]. Under long-day conditions, *TSF* and *FT* expression is up-regulated in phloem companion cells in the leaves [31]. Transcripts of *TFL1*, the paralogous of an additional *Arabidopsis PEBP* gene *ARABIDOPSIS THALIANA CENTRORADIALIS* (*ATC*), are weakly accumulated in the inner cells of matures shoot meristemsin the vegetative phase, whereas the transcript level increases following the switch to flowering [32,33]. The TFL1 protein is a mobile signal that is translocated from the inner shoot meristems cells to the outer cells and coordinates shoot meristem identity [34]. With regard to flowering, *TFL1* and *FT* are functionally antagonist, because *FT* is a floral activator, whereas *TFL1* is a floral repressor, but both proteins are able to interact with FD to regulate the FD-dependent transcription targets [35]. *BROTHER OF FT AND TFL1* (*BFT*) modulates the function of the FT–FD module and may provide an adaptation strategy that fine-tunes photoperiodic flowering under high salinity [15]. *MFT* is specifically induced in the radical-hypocotyl transition zone of the embryo in response to ABA, and *mft* loss-of-function mutants show hypersensitivity to ABA in seed germination. In germinating seeds, *MFT* expression is directly regulated by transcription factors *ABA-INSENSITIVE3* (*ABI3*) and *ABI5* in the ABA signaling pathway. In addition, *MFT* promotes embryo growth via a negative feedback loop by directly repressing *ABI5* expression in the ABA signaling pathway during seed germination [36].

The *Gossypium* genus comprises more than 50 species and includes many important cotton species [37]. Cotton is one of the most important natural textile fiber crops. The seeds are also a source of oil and protein meal. Novel insights into *Gossypium* biology have been provided by whole-genome sequencing of *Gossypium raimondii* U., *Gossypium arboretum* L. and *G*. *hirsutum* [38–40]. Most of studies of *Gossypium* species have focused on the allopolyploids *Gossypium hirsutum* and *Gossypium barbadense* (2n = 52) [39,41–44], and the diploids *Gossypium arboreum* and *Gossypium raimondii* (2n = 26) [40,45]. *Gossypium hirsutum* is a tetraploid species considered to have originated through hybridization of the D-genome species *G*. *raimondii* and the A-genome species *G*. *arboreum*. Meanwhile, *Gossypium hirsutum* consists of semi-wild races and domesticated cultivars adapted to diverse geographical and ecological environments and has a long evolutionary history [46]. Cultivated upland cotton consists of several semi-wild races, such as ‘marie-galante’, ‘paimerii’, ‘morrilli’, ‘punctatum’, ‘yucatanense’, ‘richmondii’ and ‘latifolium’. Semi-wild upland cotton shows superior resistance to insects, drought and disease than cultivated germplasm. In addition, the semi-wild race and cultivated upland cotton differ in photoperiod sensitivity. The semi-wild races flower only under short-day conditions, whereas flowering of cultivated upland cotton is insensitive to day length. In order to study the flowering-related genes in cotton, we applied different photoperiod treatments to selected semi-wild and cultivated genotypes of upland cotton. The aim of this study was to analyze genotype- and maturity-specific expression of *PEBP*-like genes in upland cotton germplasm and undertake ectopic transgenic analysis of *GhFT* and *GhPEBP2* genes and their promoters. We demonstrated the interaction of two PEBP-like proteins and the FD-like bZIP transcription factor GhFD, which is conserved in *Arabidopsis* and other species. This work highlights the involvement of *PEBP*-like genes in photoperiod response and regulation of flowering time in different cotton genotypes, and contributes to an improved understanding of the function of *PEBP*-like genes in cotton.

## Materials and Methods

### Plant materials and growth conditions

Seeds of the upland cotton Semi-wild race ‘latifolium’ were abtained from the National Wild Cotton Nursery, Sanya, China. Samples were collected from plants of the three early-flowering cultivars ‘CCRI36’, ‘CCRI74’ and ‘CCRI50’ and three late-flowering cultivars ‘CCRI41’, ‘CCRI60’ and ‘Lu28’ of upland cotton cultivated on the experimental farm of the Cotton Research Institute of Chinese Academy of Agricultural Sciences (CAAS), Anyang, Henan. All plants were grown in the field on the experimental farm of Anyang. We sampled roots, stems, leaves and shoot apicals with two fully expanded leaves stage, buds at 5mm in length, flowers at anthesis and fibers 10 days after flowering from plants of the cultivar ‘CCRI36’. We collected leaves and shoot apicals samples from two fully expanded cotyledons to four fully expanded leaves at 08:30 in the early- and late-maturing cultivars experiment.

Plants of the semi-wild race ‘latifolium’ and the cultivar ‘CCRI36’ subjected to different photoperiod treatments were grown initially in the phytotron under long days (14/10 h light/dark photoperiod, 08:00–22:00 light). Half of the plants were treated with short day (10/14 h light/dark, 08:00–18:00 light) once the plants had developed two fully expanded leaves. In the phytotron, the seeds germinated and the cotyledons were fully expanded after about seven days, and the seedlings developed new leaves every six days. We collected leaves and shoot apicals samples from two fully expanded cotyledons to five fully expanded leaves at 08:30.Wild type *Arabidopsis thaliana* accession Colombia 0 (Col-0) plants was grown in the greenhouse at 22°C under long days (16/8 h light/dark photoperiod).

### Gene cloning, vector construction and transformation

For nine *GhPEBP*-like genes, we designed specific primers to amplify the open reading frame using cDNA templates prepared from different tissues of CCRI36. For the *GhFT* and *GhPEBP2* genes, we designed a pair of infusion primers to amplify the open reading frame and cloned to pBI121vector using XbaI and SacI enzyme sites. We also designed a pair of infusion primers to amplify the promoters of *GhFT* and *GhPEBP2* sequences from the genomic DNA of CCRI36 and cloned to pBI121vector using HindIII and XbaI enzyme sites. PrimeSTAR^®^ GXL DNA polymerase (TaKaRa Tokyo, Japan) was used to amplify the two genes with the following cycling profile: 98°C for 1 min, and 30 cycles of 98°C for 10 s, 55°C for 15 s, and 68°C for 1 min. The amplified products were cloned into vector pBI121 (Clontech, Palo Alto, CA, USA) and sequenced from both ends. *Arabidopsis* plants were transformed using the *Agrobacterium*-mediated gene transfer method described previously [47]. Transgenic *Arabidopsis* plants were selected with kanamycin. Flowering date, the number of rosette leaves, and the number of cauline leaves on the main inflorescence of individual plants were recorded for transgenic lines in the T_3_ generation. Samples for quantitative real-time PCR (qRT-PCR) were harvested 14 days after sowing (DAS). Statistical test was used by one-way analysis of variance Duncan’s method.

### Sequence alignment and phylogenetic analysis

The amino acid sequences of the proteins analyzed in this study were downloaded from GenBank. Accession numbers of all species are listed in S1 Table. Multiple sequence alignment was performed with ClustalW (http://www.ebi.ac.uk). A phylogenetic tree was constructed using the neighbor joining method with Molecular Evolutionary Genetics Analysis (MEGA) software MEGA5.05 [48]. Branch support was estimated using bootstrapping with 1000 replicates.

### Protein structure prediction and promoter analysis

Protein structure prediction was performed using SWISSMODEL (http://swissmodel.expasy.org/interactive). Promoter analysis was performed with the software PlantCARE (http://bioinformatics.psb.ugent.be/webtools/plantcare/html/) and PLACE (http://www.dna.affrc.go.jp/htdocs/PLACE/).

### Quantitative Real-Time PCR

Total RNA was isolated from samples using a plant RNA purification kit (Tiangen, Beijing, China). Reverse transcription-PCR was carried out using a SuperScript™ III First-Stand Synthesis System for RT-PCR (Invitrogen, Carlsbad, USA). Transcript levels were then determined by qRT-PCR using the 7500 Real-Time PCR System (Applied Biosystems, Foster City, CA, USA) and SYBR Premix Ex Taq (2×) (TaKaRa). Gene-specific primer pairs used for the PCR amplifications are listed in S2 Table. To normalize variance among samples, *actin* was used as an endogenous control. Determination of reaction specificities and data processing were performed as described previously [49]. Three biological replicates were analyzed. The data were analyzed with Graph Pad Prism 5.

### β-glucuronidase staining

Tissue samples of *Arabidopsis* wild type Col-0 and transgenic plants were incubated in staining solution (0.1M NaPO_4_ pH 7.0, 10.0mM EDTA, 0.1% Triton X-100, 1.0mM K_3_Fe(CN)_6_, 2.0mM X-Gluc) overnight at 37°C. The stained samples were washed several times with 50% ethanol until the tissue was clears, with incubation for approximately 12 h between each change in 50% ethanol [50].

### Yeast two-hybrid and bimolecular fluorescence complementation analysis

The full-length sequence of *GhFD* was amplified and cloned into pGBKT7 vector, a bait protein is expressed as a fusion to the Gal4 DNA-binding domain (DNA-BD). Tested for autoactivation activity and toxicity were conducted. In addition, *GhFT* and *GhPEBP2* were amplified by PCR and cloned into the pGADT7 vector, prey proteins are expressed as fusions to the Gal4 activation domain (AD) (Clontech, USA). The resulting recombinant plasmid pGBKT7-GhFD was introduced into yeast strain Y2H, and the recombinant plasmids pGADT7-GhFT and pGADT7-GhPEBP2 were introduced into yeast strain Y187 respectively. When bait and prey fusion proteins interact, the DNA-BD and AD are brought into proximity to activate transcription of four reporter genes. Additionally, *GhFD* was also cloned into pGADT7 vector, and *GhFT* / *GhPEBP2* were cloned into the pGBKT7 vector respectively. Two-hybrid interactions were assayed on selective SD/-Trp/-Leu (DDO) and SD/-Ade/-His/-Leu/-Trp (QDO) media supplemented with X-α-Gal and Aureobasidin A.

To measure in vivo interactions, open reading frames of full-length *GhFD*, *GhFT* and *GhPEBP2* genes coding sequences were cloned into vectors pSPYNE and pSPYCE, which contain DNA encoding the N-terminal and C-terminal regions of yellow fluorescent protein, respectively. Molecular techniques of BiFC were performed using protocols according to [51,52]. Fully-expanded rosette leaves of Arabidopsis Col-0 plants grown for 4 weeks under short-day conditions were collected for the protoplast isolation. Protoplasts was transformed with 10μg of plasmid DNA and incubated at 22°C. Protoplasts isolated from *Arabidopsis* leaves were transformed with the following combinations of plasmids: pSPYNE-GhFD, pSPYNE-GhFT, pSPYNE-GhPEBP2, pSPYCE-GhFD, pSPYCE-GhFT and pSPYCE-GhPEBP2. The samples were observed with a FV1000 confocal laser scanning microscope (Olympus, Tokyo, Japan).

## Results

### Identification of *PEBP*-like genes in *Gossypium hirsutum*

To identify *PEBP*-like family genes in upland cotton, we searched databases containing genomic sequences for *G*. *raimondii*, *G*. *arboreum* and *G*. *hirsutum* and identified nine putative *GhPEBP*-like genes (Table 1). We then amplified cDNAs based on the annotated coding sequences and compared their structures. Analysis of genomic sequences revealed that the aforementioned seven genes conserved the characteristic genomic organization, containing four exons and three introns in identical positions, and the latter two genes showed a different structure with two exons and one intron (S1 Fig). Comparisons of amino acid sequences with FT/TFL1-like sequences from *Arabidopsis*, in conjunction with phylogenetic analysis of the multiple sequence alignment (see below), enabled interpretation of the cloned sequences as either a FT orthologue, MFT orthologue, TFL1 orthologue and PEBP orthologue. Seven *FT*/*MFT*/*TFL1* genes were shown similarity with the flowering plant species *PEBP* family, and two PEBP sequences showed conserved with *PEBP* genes of bacteria and archaea. Based on the observed sequence similarities the genes were respectively designated as *GhFT*, *GhMFT1/2*, *GhTFL1a/1b/1c/1d* and *GhPEBP1/2*.

**Table 1:** Information of genes in different genomes.

Gene name	Chr^D^	Locus in D genomic	Chr^A^	Locus in A genomic	Chr^AD^	Locus in AD genomic
GhFT	Chr04	Gorai.004G264600.1	Ca7	Cotton_A_05804	scaffold246.1/Dt_chr5	CotAD_14755/CotAD_04102
GhTFL1a	Chr07	Gorai.007G010800.1	Ca10	Cotton_A_07540	At_chr7/Dt_chr9	CotAD_02721
GhTFL1b	Chr06	Gorai.006G155800.1	Ca10	Cotton_A_13428	At_chr11/Dt_chr5	CotAD_43979/CotAD_02907
GhTFL1c	Chr09	Gorai.009G403800.1	Ca2	Cotton_A_31651	At_chr11	CotAD_37875
GhTFL1d	Chr01	Gorai.001G121800.1	Ca1	Cotton_A_09584	Dt_chr1/Dt_chr13	CotAD_15834
GhMFT1	Chr09	Gorai.009G174600.3	Ca6	Cotton_A_04728	At_chr7/scaffold2081.1	CotAD_55039/CotAD_41263
GhMFT2	Chr06	Gorai.006G192300.1	Ca10	Cotton_A_13046	scaffold4006.1/Dt_chr6	CotAD_03154/CotAD_70215
GhPEBP1	Chr02	Gorai.002G264900.1	Ca12	Cotton_A_00307	At_chr9/scaffold2318.1	CotAD_03575
GhPEBP2	Chr12	Gorai.012G117400.1	Ca2	Cotton_A_03455	At_chr12/Dt_chr12	CotAD_67783

Abbreviations are as follows: Chr, chromosome; A, D and AD, *Gossypium arboreum*, *Gossypium raimondii* and *Gossypium hirsutum* genomes. Chr and identity indicate the chromosomal location and identity of a given gene. AD indicate the *G*. *hirsutum* genome references from BGI-CGP.

### Phylogenetic relationships of the *PEBP*-like genes

**Fig 1:**
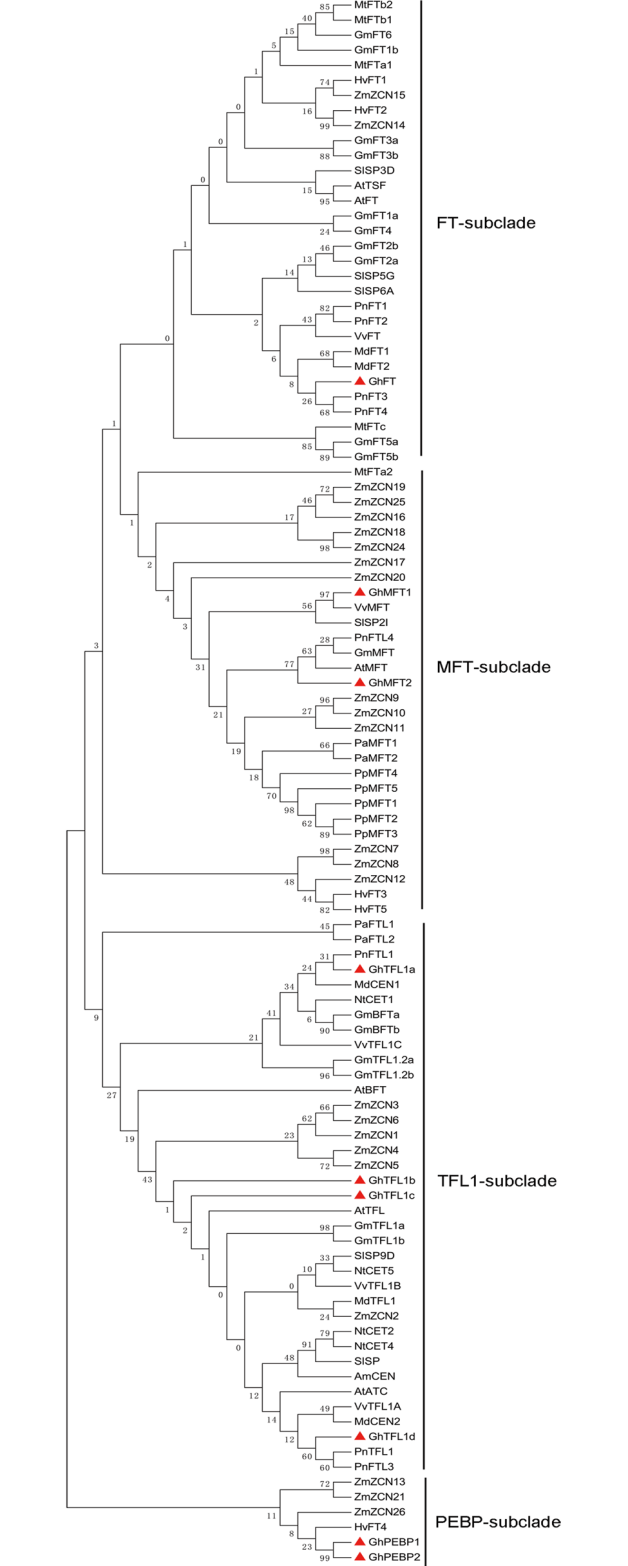
Phylogenetic analysis of *PEBP* family members of *Gossypium hirsutum* and other plants species. The unrooted phylogenetic tree was constructed using the neighbor-joining method from protein sequences from *Physcomitrella patens* (*PpPEBP*), *Picea abies* (*PaPEBP*), *Antirrhinum majus* (*AmPEBP*), *Populus nigra* (*PnPEBP*), *Arabidopsis thaliana* (*AtPEBP*), *Vitis vinifera* (*VvPEBP*), *Solanum lycopersicum* (*SlPEBP*), *Malus* × *domestica* (*MdPEBP*), *Medicago truncatula* (*MtPEBP*), *Glycine max* (*GmPEBP*), *Gossypium hirsutum* (*GhPEBP*), *Nicotiana tabacum* (*NtPEBP*), *Zea mays* (*ZmPEBP*) and *Hordeum vulgare* (*HvPEBP*). The data sources for all *PEBP*s are listed in S1 Table. The red triangle indicates the upland cotton *PEBP*-like genes.

To study the phylogenetic relationships of *GhPEBP*-like genes and other *PEBP* homologues, we compiled a data set of 104 PEBP amino acid sequences from 14 species, comprising a bryophyte (*Physcomitrella patens* H.), a gymnosperm (*Picea abies* L.) and angiosperms (*Antirrhinum majus* L., *Populus nigra* L., *Arabidopsis thaliana* L., *Vitis vinifera* L., *Solanum lycopersicum* L., *Malus domestica* B., *Medicago truncatula* G., *Glycine max* L., *Nicotiana tabacum* L., *Gossypium hirsutum* L., *Zea mays* L. and *Hordeum vulgare* L.). An unrooted phylogenetic tree derived from the amino acid sequences was constructed using the neighbor-joining method and consisted of four subclades (Fig 1). The FT-subclade comprised GhFT, *Arabidopsis* FT and TSF, as well as other FT orthologous proteins identified in other species. GhFT displayed the characteristic features of the FT protein subclade, which include conservation of amino acid residues Tyr85 and Gln139 (S1 Fig). The second distinctive monophyletic clade consisted of the two GhMFT proteins, *Arabidopsis* MFT and putative orthologous proteins identified in other dicotyledons. The third subclade contained the four GhTFL1 proteins, *Arabidopsis* BFT, TFL1 and ATC. The TFL1 proteins shared conserved amino acid residues His88 andAsp144 in similar positions with *Arabidopsis* TFL1. The fourth subclade consisted of proteins that contained the conserved PEBP domain with a structure specific to bacteria and archaea, but not a eukaryote-specific sequence identified in previous plant studies. Most GhPEBP-like proteins exhibited a closer relationship to PEBP proteins from other dicotyledons than to those from monocotyledons. The gymnosperm *Picea abies* had MFT subclade and TFL1 subclade, and the bryophyte *Physcomitrella patens* only had MFT subclade. These findings indicated that PEBP proteins may be highly conserved in different species. Protein structure analysis is also predicted that GhFT and GhTFL1 proteins were structurally similar to its homologous proteins of *Arabidopsis* and rice [14,28], whereas GhPEBP-like proteins was more similar to predicted PEBP protein of *Escherichia coli* M. [53] (S2 Fig).

### Tissue-specific expression of *GhPEBP*-like genes in *Gossypium hirsutum*

**Fig 2:**
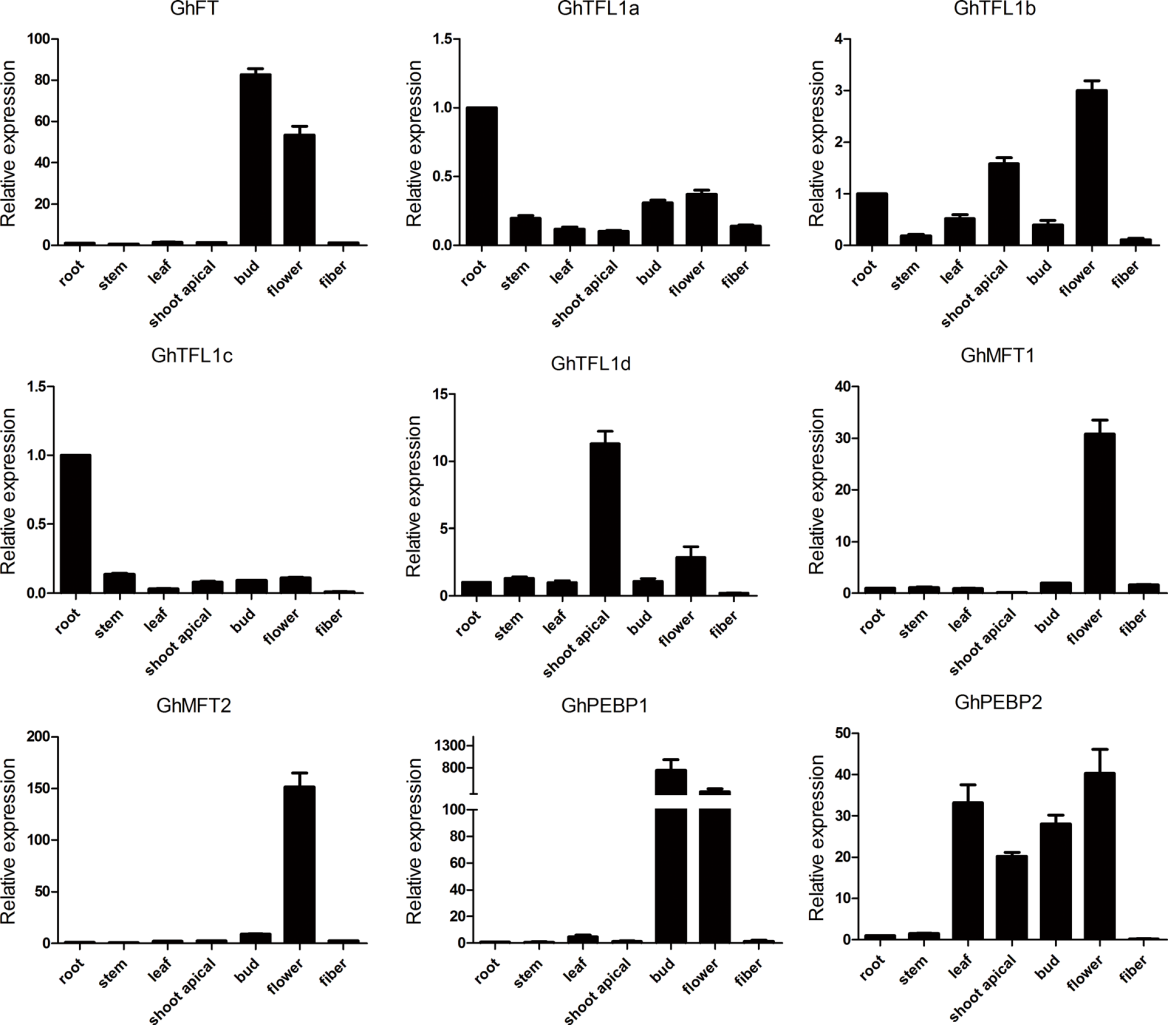
Tissue-specific expression patterns of *GhPEBP*-like genes. qRT-PCR of *GhPEBP*-like in seven tissues of upland cotton ‘CCRI36’. The roots, stems, leaves and shoot apicals with two fully expanded leaves stage, buds at 5mm in length, flowers at anthesis and fibers 10 days after flowering from plants were collected from the cultivar ‘CCRI36’. Values have been normalized to the transcript level of the *ACTIN* gene.

*GhPEBP*-like transcription levels were examined by quantitative real-time PCR (qRT-PCR) in samples of roots, stems, leaves, shoot apicals, buds, flowers and fibers of the short-season Chinese upland cotton cultivar ‘CCRI36’. All the samples were collected from plants growing on the farm of Anyang. To differentiate individual *GhPEBP*-like genes, we designed gene-specific primers by avoiding highly conserved sequence regions. Transcript levels for *GhFT* and *GhPEBP1* were higher in buds and flowers compared with vegetative organs (Fig 2). Accumulation of *GhTFL1a* and *GhTFL1c* transcripts was highest in roots, whereas *GhTFL1b* and *GhTFL1d* transcript levels were highest in shoot apicals and flowers. *GhMFT1* and *GhMFT2* were transcribed mainly in flowers with low transcript levels observed in other tissues. The highest levels of *GhPEBP2* transcripts were detected in leaves, shoot apicals, buds and flowers.

### Expression of *GhPEBP*-like genes under different photoperiod treatments

**Fig 3:**
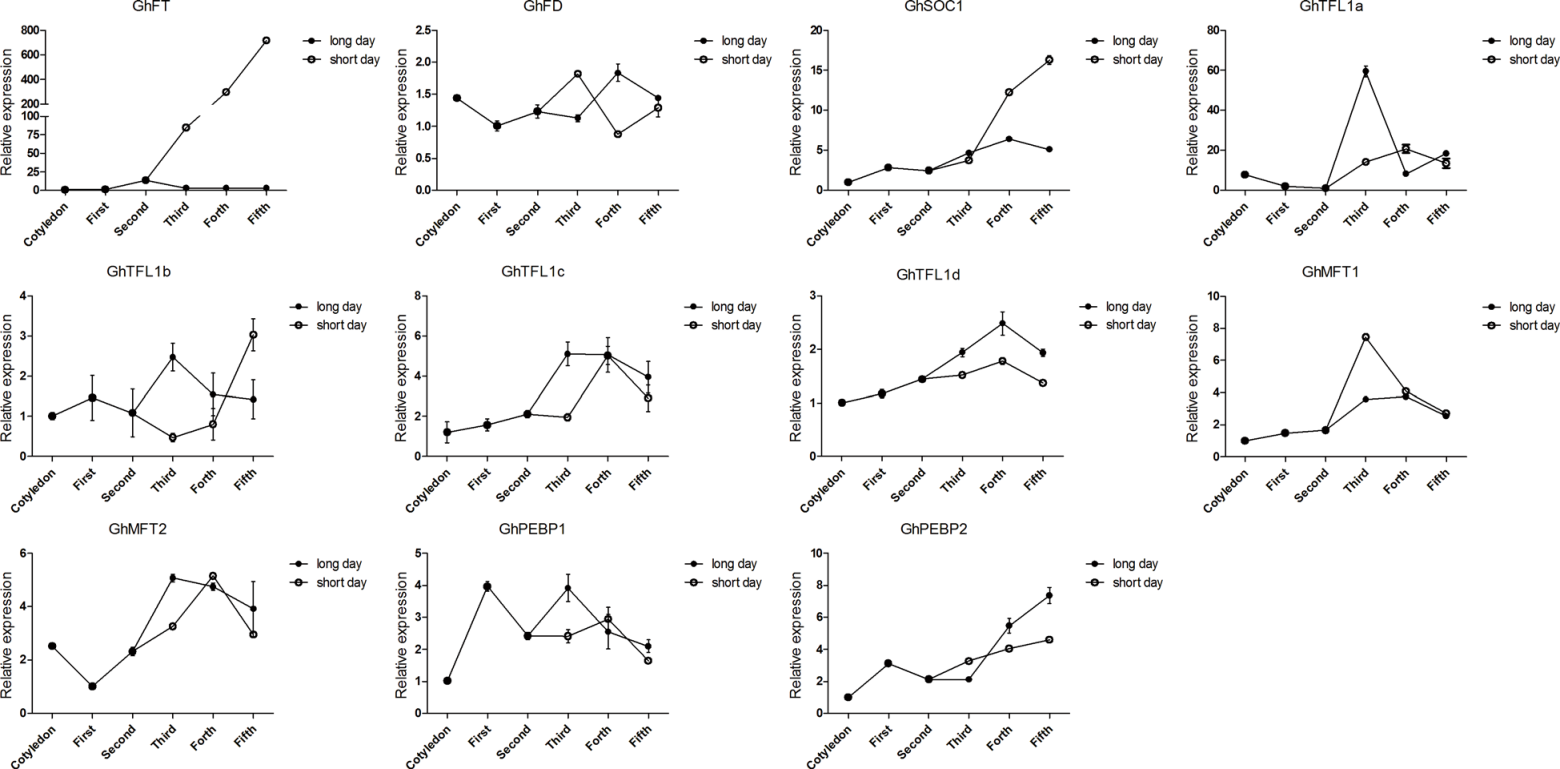
Expression patterns of *PEBP*-like genes in the upland cotton semi-wild race ‘latifolium’. Plants were initially grown from seed germination under long-day conditions. Half of the plants were transferred to short-day conditions at the two fully expanded leaves stage. Cotyledons indicated two fully expanded cotyledons. First to Fifth indicated first fully expanded leaves to fifth fully expanded leaves.

**Fig 4:**
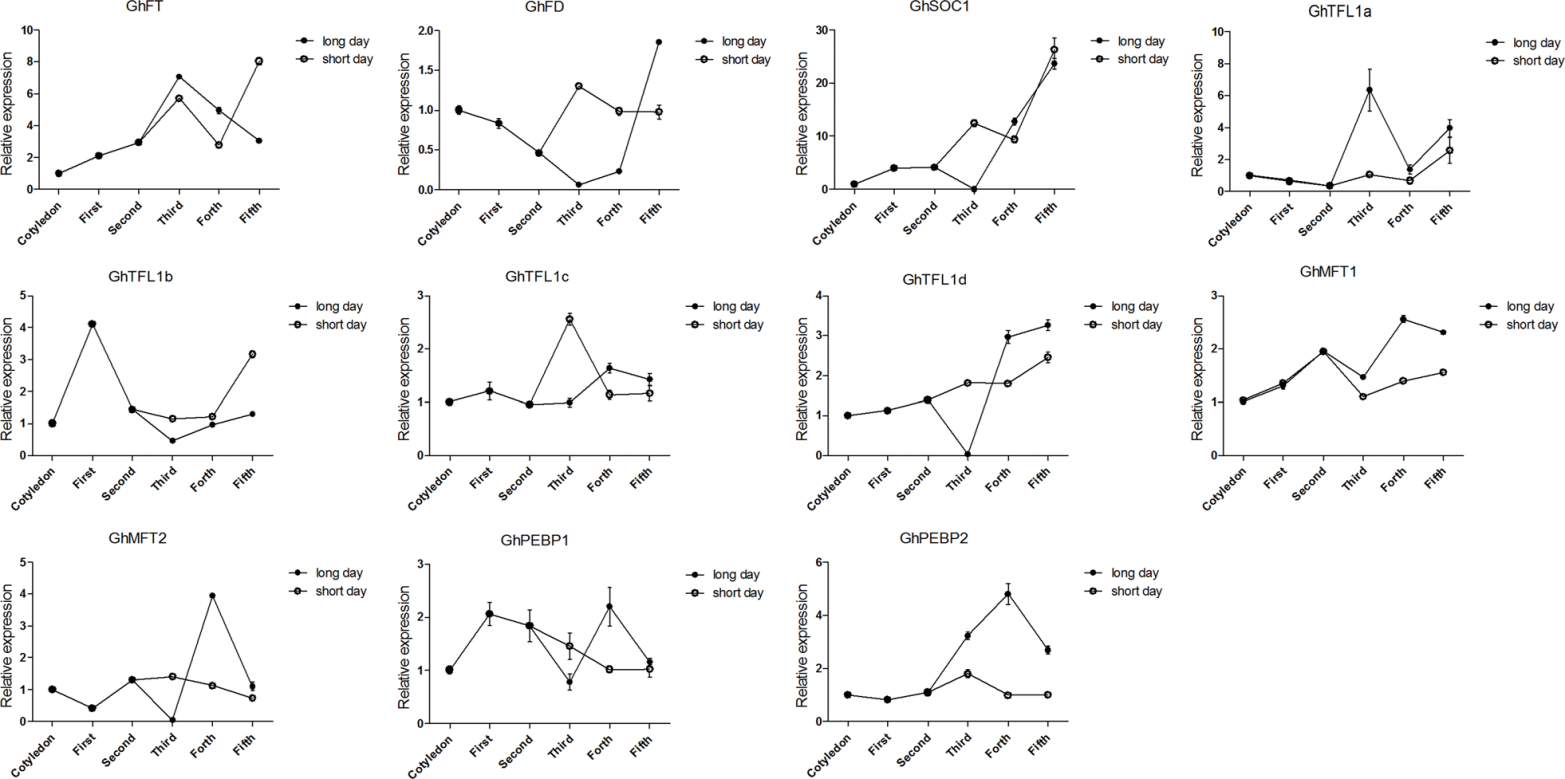
Cotton *PEBP*-like genes show different expression patterns in cultivated cotton ‘CCRI36’. Plants were initially grown from seed germination under long-day conditions. Half of the plants were transferred to short-day conditions at the two fully expanded leaves stage. Cotyledons indicated two fully expanded cotyledons. First to Fifth indicated first fully expanded leaves to fifth fully expanded leaves.

Due to the different photoperiod sensitivities of semi-wild races and cultivars of upland cotton, we selected the semi-wild race ‘latifolium’ and cultivar ‘CCRI36’ as representative genotypes to analyze *GhPEBP*-like expression patterns under different photoperiod treatment. Interestingly, *PEBP*-like genes showed different regulation patterns with respect to both long day and short day conditions and genotypic differences. According to previous studies of other plant species, *SOC1* and *FD* are important flowering-related genes [54,55]. Thus, we also chose the two homologous genes *GhSOC1* (AEA29618.1) and *GhFD* to investigate the expression pattern in upland cotton. The gene expression levels were determined in the whole aboveground portion of the harvested plants. For semi-wild race, relative expression level of *GhFT*, *GhSOC1* and *GhMFT1* was strongly increased, whereas *GhPEBP2* and *GhTFL1d* expression was decreased, under short days compared with long days, and *GhFT* especially showed hundreds of fold enrichment (Fig 3). The other identified genes were not unambiguously detected under both long- and short-day conditions. For the cultivar ‘CCRI36’, *GhFT*, *GhMFT1*, *GhPEBP2* and *GhTFL1a* expression were increased under long days relative to that under short days. *GhTFL1b* showed the strongest expression induction and was increased under short days (Fig 4). It would be interesting in the future to examine in more detail the expression of *PEBP*-like genes, e.g. in leaves and shoot apical meristems, in diverse upland cotton genotypes.

### Expression of flowering-related genes in different maturing upland cotton cultivars

**Fig 5:**
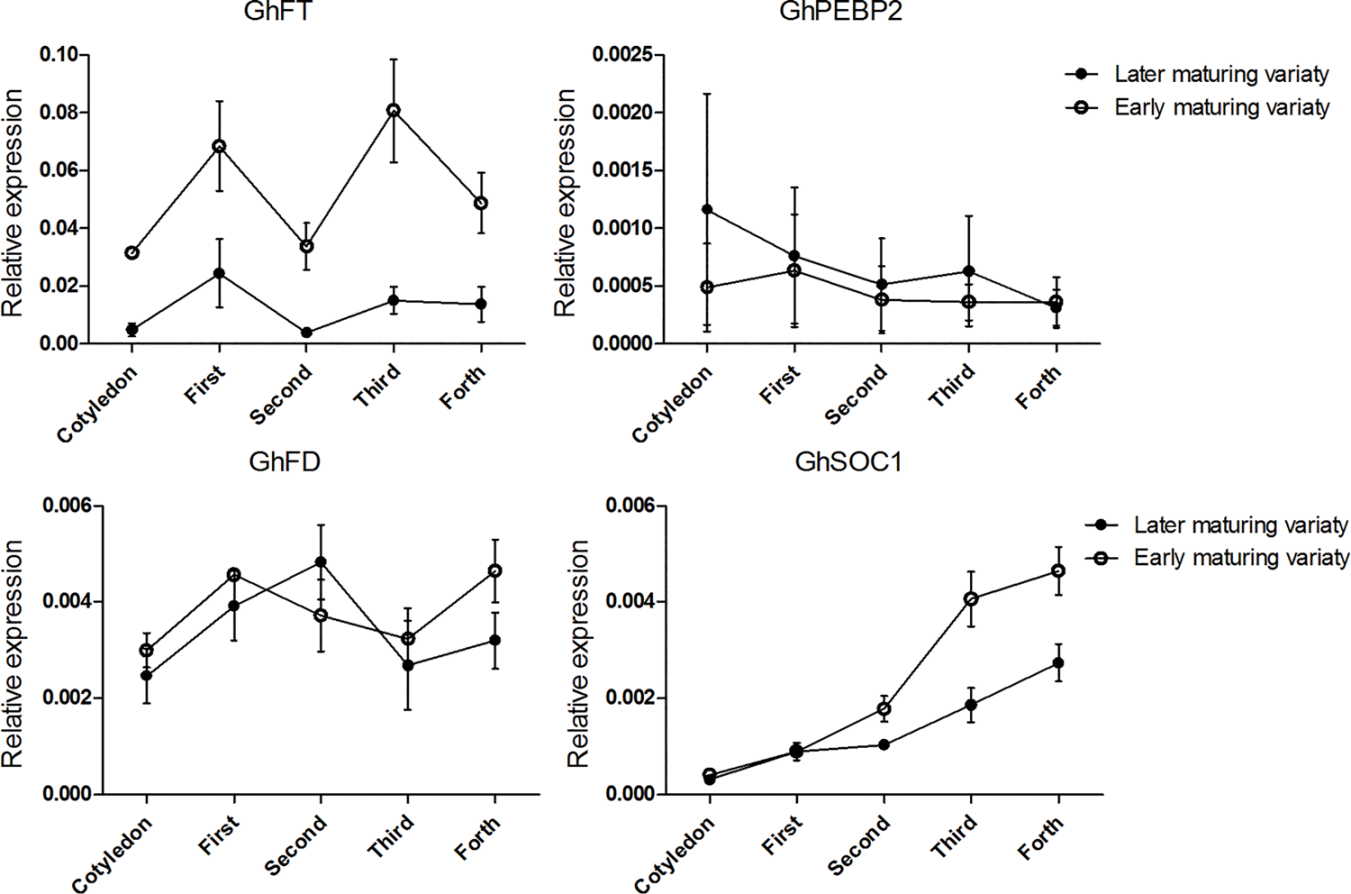
Expression of flowering-related genes in early- and late-maturing cultivars of upland cottons. The growth period of earlier-maturing cultivars (‘CCRI36’, ‘CCRI74’ and ‘CCRI50’) was 102~106 days, and that of later-maturing cultivars (‘CCRI41’, ‘CCRI60’ and ‘Lu28’) was 123~138 days. Cotyledons indicated two fully expanded cotyledons. First to Forth indicated first fully expanded leaves to forth fully expanded leaves. The error bar indicated expression levels of three cultivars.

Although previous studies have described flowering-related genes of *G*. *hirsutum*, these studies have reported tissue-specific expression in a single cultivar [56–59] or in different *Gossypium* species such as *G*. *hirsutum* and *G*. *arboreum* [60]. The relative expression of flowering-related genes in early- and late-maturing cultivars of upland cotton has not been investigated previously. Thus, we tested the expression patterns of *GhFT*, *GhPEBP2*, *GhFD* and *GhSOC1* genes in whole aboveground tissues of early- and late-maturing cultivars from the cotyledon to the four-leaf stages. As shown in Fig 5, *GhFT* transcript levels were higher in early-maturing cultivars than in late-maturing cultivars throughout the experimental period. *GhSOC1* showed the strongest induction, with transcript levels increasing gradually from the two fully expanded leaves stage, and expression was higher in early-maturing cultivars. Neither *GhPEBP2* nor *GhFD* transcript levels showed discernible variation in the aboveground tissues of plants of either maturity class.

### Ectopic expression and promoter analysis of *GhFT* and *GhPEBP2*

**Fig 6:**
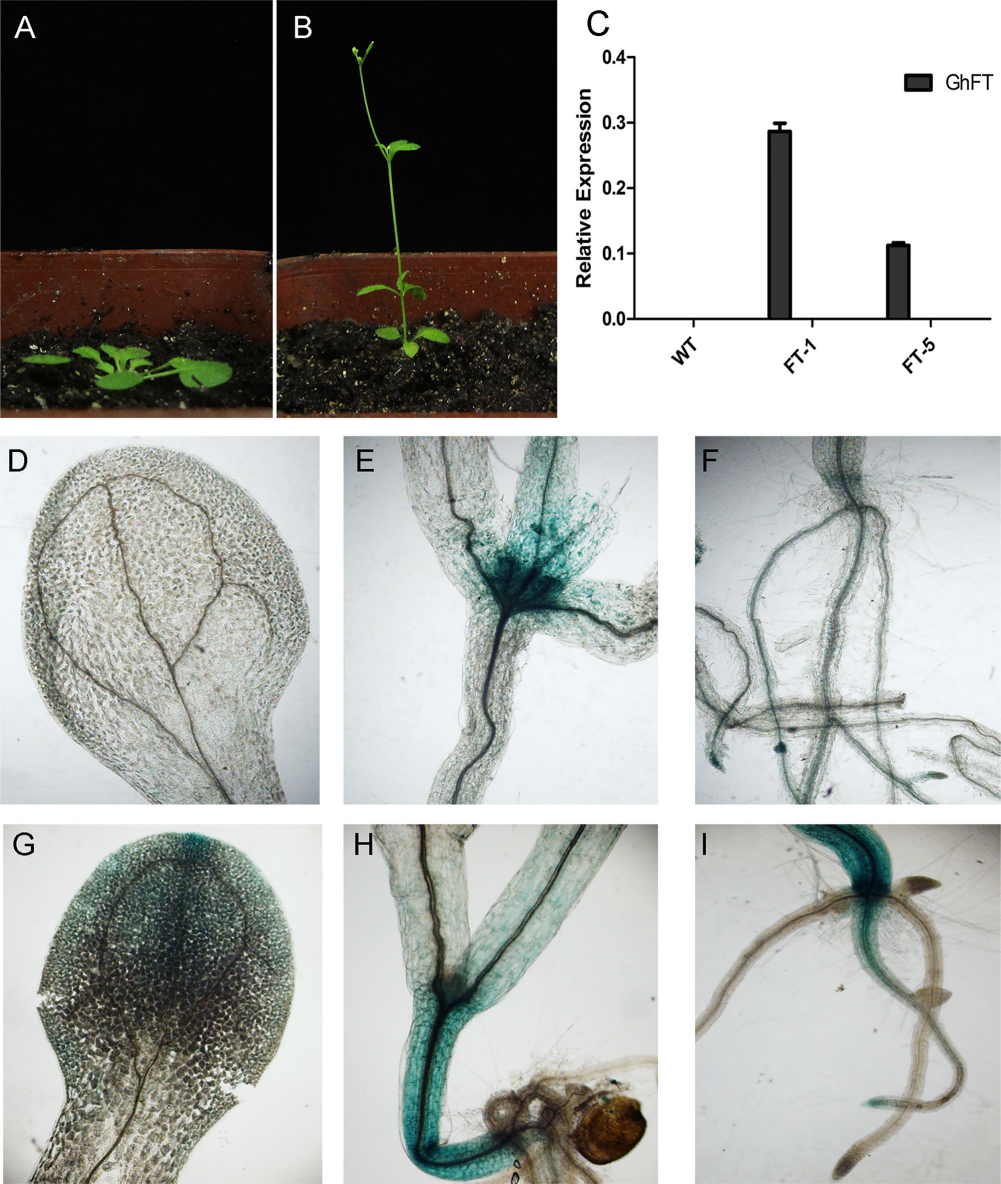
Analysis of *GhFT* and *GhPEBP2* expression. **(A)** Wild type and **(B)** transgenic *GhFT Arabidopsis* plant. **(C)** Transcript levels of *GhFT* in different transgenic lines. **(D**-**I)** Promoter analysis of *GhFT* and *GhPEBP2* in *Arabidopsis*. The cotyledon (**D**), shoot apical and stem (**E**), and root (**F**) of transgenic *pGhFT*::*GUS* plants. The cotyledon (**G**), shoot apical and stem (**H**), and root (**I**) of transgenic *pPEBP2*::*GUS* plants.

To confirm the functional roles of *GhFT* and *GhPEBP2* in the regulation of flowering time, we constructed an overexpression vector using the *cauliflower mosaic virus* 35S promoter to drive constitutive expression of the genes in wild-type *Arabidopsis* plants. Five transgenic *Arabidopsis* lines were obtained and qPCR confirmed that the *GhFT* and *GhPEBP2* were successfully integrated into the *Arabidopsis* genome using the 35S forward primer and a gene-specific reverse primer. Flowering dates for two T3 transgenic lines indicated that *GhFT* significantly promoted flowering on average by about 3.59 days under long days and 17.53 days under short days (Fig 6A–6C, Table 2). These changes in flowering dates were statistically significant. This precocious flowering was correlated with a decrease in the number of rosette leaves and an increase in the number of cauline leaves of individual plants. Because overexpression of *GhFT* resulted in promotion of flowering, we analyzed the expressions of *Arabidopsis* genes that regulate flowering time. This analysis revealed that *AtFT* was significantly promoted, whereas expression of the floral-organ-related genes *AtAP1*, *AtLFY* and *AtSPL3* decreased in *35S*::*GhFT* plants (S3 Fig). In contrast, the three *GhPEBP2* transgenic lines showed inconsistent phenotypic and flowering trends, with flowering promoted in one line and delayed in two lines (Table 2, S4 Fig). This result indicated that *GhFT* can promote flowering under both long- and short-day condition, whereas *GhPEBP2* did not affect flowering consistently.

**Table 2:** Comparison of days of anthesis under different photoperiod conditions.

Genotype^a^	Anthesis(DAS)^b^	Number of rosette leaves^c^	Number of cauline leaves^d^	Number of plants
LD condition				
*Col-0*	28.53±1.125	8.93±1.387	3.67±0.488	19
*35S*::*GhFT*	24.94±0.826**	5.42±2.020**	7.86±2.958**	41
SD condition				
*Col-0*	47.12±4.372	23.65±4.867	4.06±0.622	17
*35S*::*GhFT*	29.59±2.062**	5.59±2.175**	6.86±2.031**	35
LD condition				
*Col-0*	34.00±2.03	12.80±1.77	3.30±0.57	20
*35S*::*GhPEBP2-2*	36.53±2.69**	13.53±2.53	3.07±0.70	15
*35S*::*GhPEBP2-3*	35.48±2.35**	12.46±1.32	2.96±0.81	24
*35S*::*GhPEBP2-5*	31.54±1.48**	9.46±1.45**	2.39±0.74**	28

^a^ Genetic background: Col, Columbia; transgenic lines of *GhFT* under different photoperiod conditions.

^b^ Indicators of anthesis (days after sowing (DAS)), shown as average ± standard deviation (SD)

^c,d^ Indicators of leaf numbers, shown as average ± SD. Plants were grown under long days (16 h/8 h photoperiod) and short days (8 h/16 h photoperiod).

** Values significantly different from Col-0 at *p* < 0.01.

In addition, we analyzed the *GhFT* and *GhPEBP2* promoter sequences 1500bp upstream of the ATG start codon, based on the available *Gossypium* genome sequences, using the PlantCARE and PLACE databases. Both *GhFT* and *GhPEBP2* promoters contained common elements such as a CAAT box, TATA box and many light-responsive elements, and elements such as different binding motifs specific for transcriptional factors (S5 Fig). The promoter of *GhFT* was predicted to show shoot-specific expression responsiveness, abscisic acid (ABA) and auxin responsiveness. The ABA-responsive motif, ethylene-induced motif and MYB binding site involved in drought-inducibility elements were identified in the *GhPEBP2* promoter. We also analyzed three transgenic *Arabidopsis* lines overexpressing *pGhFT*::*GUS* and *pGhPEBP2*::*GUS*. The *GhFT* promoter was most highly expressed in shoot apicals, followed by leaves and roots, but not in cotyledons and stems (Fig 6D–6F). In contrast, *GhPEBP2* promoter was mainly expressed in cotyledons, stems and the axial roots (Fig 6G–6I). These results indicated that *GhFT* may play an important role in plant development, whereas GhPEBP2 may play roles in abiotic stress response.

### GhFD interaction with cotton FT and PEBP2 proteins

**Fig 7:**
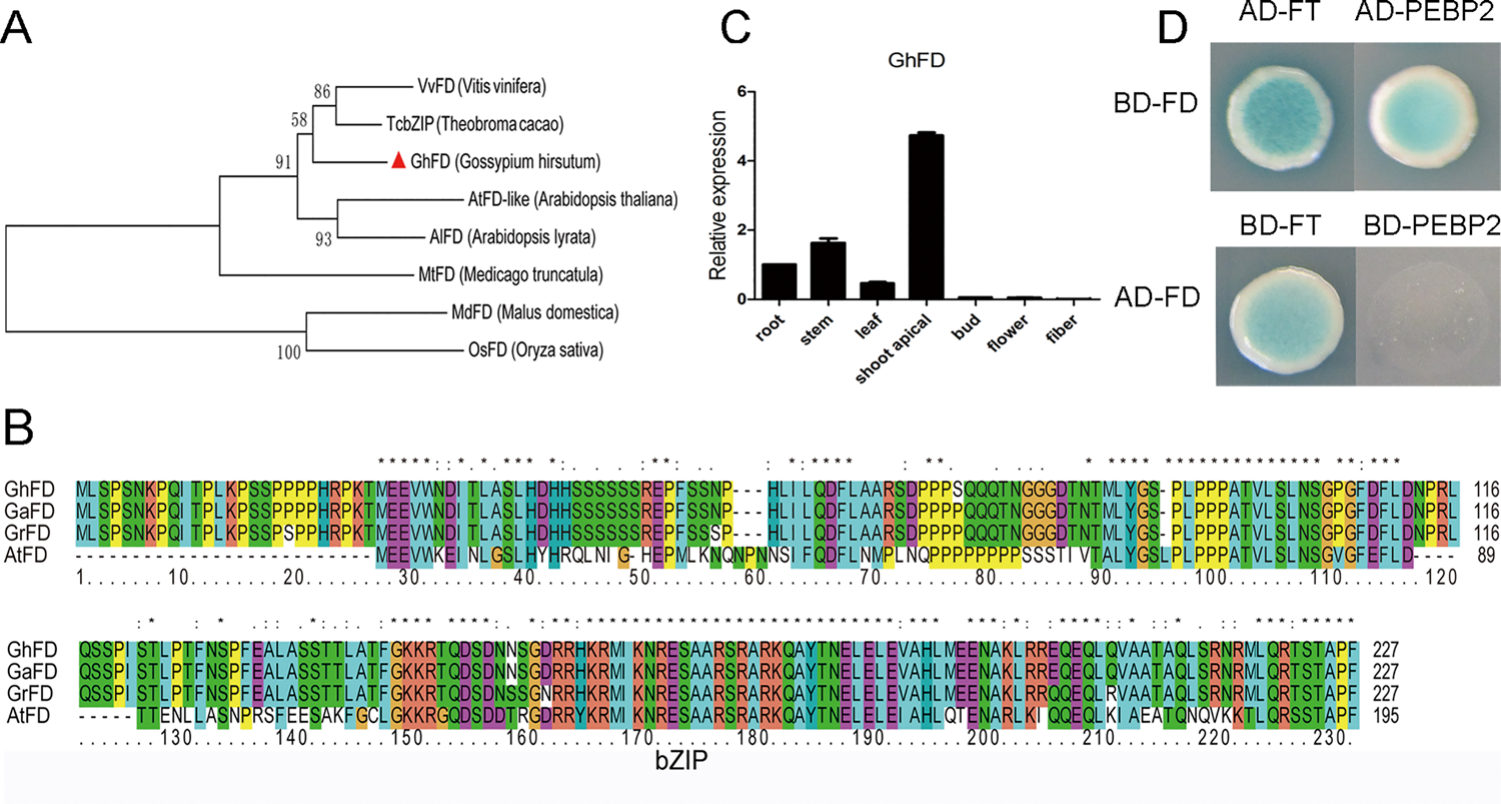
Interaction between GhFD and cotton FT and PEBP2. **(A)** Phylogenetic relationships of GhFD and FD proteins of other plant species. (**B)** Amino acid sequence alignment of GhFD proteins of *Arabidopsis* and three cotton species. The *Arabidopsis* protein sequence of FD was downloaded from The Arabidopsis Information Resource (TAIR). The three cotton FD gene sequences were obtained from respective genome sequence databases for *Gossypium raimondii* (GrFD; Gorai.003G007000.1), *G*. *arboreum* (GaFD; Cotton_A_01537_BGI-A2_v1.0) and *G*. *hirsutum* (GhFD; CotAD_70805/CotAD_02268). **(C)** Tissue-specific expression of GhFD in *G*. *hirsutum*. **(D)** Interaction of GhFD with cotton FT/PEBP2 proteins in yeast cells. The GhFD and cotton FT/PEBP2 gene was fused in-frame to the GAL4 DNA-binding domain (BD)-coding sequence and the GAL4 activation domain (AD)-coding sequences. Cell growth on Leu-Trp-His-Ade dropout selective medium (-QDO) represents positive interactions.

FT can interact with the bZIP transcription factor FD in the shoot apical meristem, in which the resultant FD–FT complex is essential for *AP1* and *LFY* induction to promote the flowering transition. In our cotton cDNA database [61], we identified a sequence in shoot apicals encoding a predicted protein that showed high similarity to *Arabidopsis FD* (Fig 7A and 7B) and contains the conserved bZIP domain and C-terminal SAP (Ser-Ala-Pro) motif (Fig 7B). RT-PCR analysis indicated that *GhFD* showed a higher expression level in shoot apicals than in other organs (Fig 7C). In order to investigate the interaction of GhFD and cotton FT and PEBP2, we designed a yeast two-hybrid assay. We first tested the autoactivation activity and toxicity of GhFD and determined that this protein exhibited autoactivation activity. Then, we cloned *GhFT* and *GhPEBP2* genes into pGBKT7 vector respectively and investigated the interaction of these two proteins and GhFD in yeast cells, which were grown on selective media with Aureobasidin A and 5-bromo-4-chloro-3-indolyl-α-D-galactopyranoside (X-α-gal). The assay indicated that GhFD could interact with GhFT protein but not with GhPEBP2 (Fig 7D).

**Fig 8:**
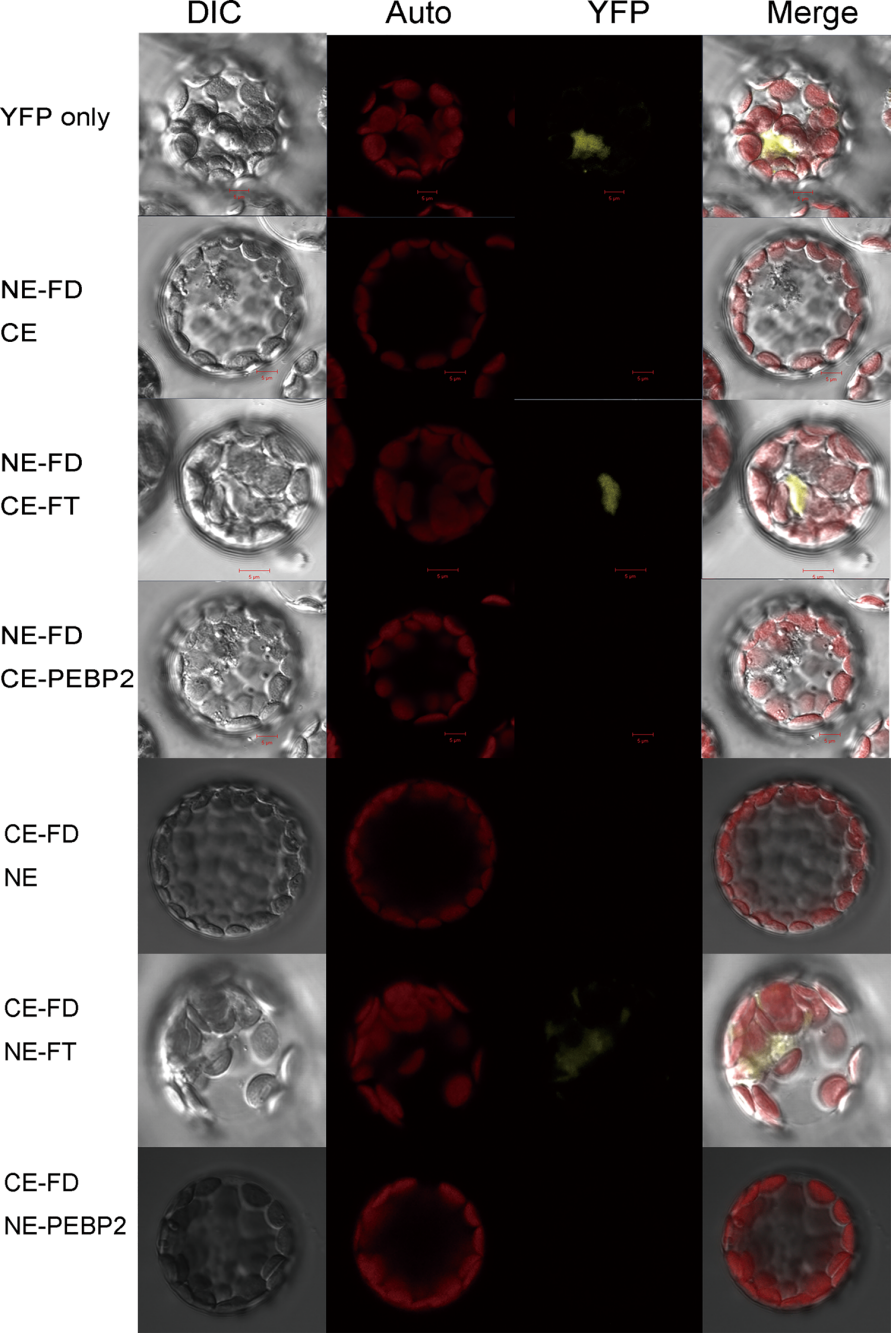
Bimolecular fluorescence complementation (BiFC) assay. The pSPYNE-GhFD/GhFT/GhPEBP2 and pSPYCE-GhFD/GhFT/GhPEBP2 constructs were transiently co-expressed in *Arabidopsis* protoplasts and visualized by differential interference contrast (DIC) and yellow fluorescence microscopy. NE and CE indicated pSPYNE and pSPYCE, respectively. Bars = 5 μm.

We next conducted bimolecular fluorescence complementation (BiFC) assays to evaluate whether GhFD and GhFT form protein complexes in plant cells. After transformation for 12–16 h, GhFD was revealed to have interaction with GhFT (Fig 8), providing evidence for GhFD-GhFT complex interaction in plant cells.

## Discussion

With the availability of genome sequences for *G*. *raimondii*, *G*. *arboreum* and *G*. *hirsutum*, genetics and molecular functional studies on *Gossypium* have entered a new era. Previous studies have investigated fiber development, leaf and floral development, biotic and abiotic stress and so on [57,62–66]. Meanwhile, there were few studies making use of molecular marker techniques to detect QTL for cotton earliness traits, such as growth period, growth stages, node of first fruiting branch, and height of node of first fruiting branch [67–70]. In this study, *GhPEBP*-like family genes were cloned from upland cotton and shown to cluster in four subclades, namely the FT subclade, MFT subclade, TFL1 subclade and PEBP subclade. According to the chromosome locus in genomic, the *GhPEBP*-like family genes could not be anchored with cotton earliness traits QTLs in previous studies [67–70]. Compared with the *Arabidopsis FT*/*TFL1* family, both *FT* and *TSF* showed similar functions in flowering time [30,71], and some of the *FT* family genes had no *TSF* and two *FT* in other species [72]. We identified five *FT*/*TFL1*-like genes, comprising one *GhFT* and four *GhTFL1*s, in upland cotton. For *MFT*, only one gene has been identified in *Arabidopsis* and other species, whereas two *MFT* genes were identified in *G*. *hirsutum*. These results indicated that differences in gene numbers have arisen during species evolution. Meanwhile, increases or decreases in the number of homologous genes may have effects on gene expression, gene function and stoichiometric balance.

The spatiotemporal expression patterns of *PEBP* genes have been determined in many species such as *Arabidopsis*, grape, and rice [7,11,73]. However, no study of *PEBP* genes expression in semi-wild and cultivated genotypes of upland cotton has been undertaken previously. We observed that transcript levels of *GhFT* had higher transcript levels in buds and flowers than in vegetative organs. The result was very similar to the previous study and the expression pattern of *FT* in *Arabidopsis* [6,59]. Although *GhTFL1*s shared similar structures, the expression induction was also completely different. *GhTFL1a* and *GhTFL1c* were most highly expressed in roots, whereas transcript levels of *GhTFL1b* and *GhTFL1d* were highest in shoot apicals and flowers. This multiple and differently expression pattern of *GhPEBP*-like genes may also contributed to distinct gene function specifically. We then examined the contribution of *GhPEBP*-like genes to the photoperiod responsiveness in different upland cotton germplasm and in early- and late- maturing upland cotton cultivars. For semi-wild type cotton, expression levels of *GhFT*, in particular, and *GhSOC1* strongly increased under short days. This induction was photoperiod specific and together with no apparent expression trend in cultivated cotton. *GhTFL1d* and *GhPEBP2* were induced and expression levels increased in both semi-wild race and cultivated upland cotton under long days, of which *GhPEBP2* showed especially high expression enrichment. Only *GhMFT1* expression was increased in semi-wild race, whereas expression was decreased in cultivated cotton, under short days. These result indicated *GhFT* and *GhSOC1* are important flowering related genes in semi-wild upland cotton. Additionally, the function of *GhPEBP2* requires further analysis to determine whether it can promote flowering under long-day condition. Semi-wild race of upland cotton could flower only under short-day conditions, whereas cultivated upland cotton has been selected to be photoperiod-insensitive during domestication and crop evolution. Thus, the key flowering genes showed dispensable effect in the photoperiod pathway. In addition, a shift in the function of these genes over the course of evolution and selection is evident.

In addition, we investigated the expression pattern of flowering related genes in early- and late- maturing cultivars. *GhFT* and *GhSOC1* showed higher transcript levels in earlier-maturing cultivars than in late-maturing cultivars, whereas *GhFD* and *GhPEBP2* showed no significant differences. The existence of *PEBP* family members that we observed in cotton here had also been described previously. For example, Pea *FT* family genes showed different expression patterns among developmental timing, tissue specificity and photoperiod response [9]. In upland cotton, *GhSOC1* also plays an important role in promoting flowering and foral organ variations [74]. Both *GhSOC1* and *GhFT* had higher expression level in early maturing cultivars from the two fully expanded leaf stage, in which the floral bud primordia have already emerged and continued to differentiate during the third fully expanded leaf stage [58]. These result indicated that *GhFT* may be an important flowering-related gene similar to *GhSOC1*. Under long day conditions it is suggested that, although *GhFT* might play fewer roles in regulating gene expression concomitant with the long domestication of cultivated cotton from semi-wild races, it still can distinguish cultivars belonging to different maturity classes.

Studies of *Arabidopsis* and other plant species had reported the effects of *FT* family genes on flowering. The *Arabidopsis FT* gene promotes flowering in heterologous plant species, and FT protein can interact with FD to regulate other flowering related genes [75,76]. The tomato *FT* orthologue also triggers systemic signals to regulate growth and flowering [77]. In the present study, we identified two *PEBP* genes, *GhFT* and *GhPEBP2*, and transformed them into wild-type *Arabidopsis*. Ectopic expression of *GhFT* in *Arabidopsis* accelerated flowering under both long- and short-day conditions. The expression patterns observed for flowering-related genes in *Arabidopsis* demonstrated that *GhFT* could promote flowering by up-regulating the expression of *FT*. However, overexpression of *GhPEBP2* had no obvious phenotypic impact. Molecular evolution studies suggest that plasticity at exon four contributes to the divergence of *FT*-like function in floral promotion [78]. This also may explain why *GhPEBP*-like genes, which contain two exons, may not contribute to flowering. In plants, the florigen FT interacts with the bZIP transcription factor FD and promotes flowering [24,28]. Thus, we analyzed the interaction of GhFD and cotton FT and PEBP2 proteins. We demonstrated that GhFD interacted with GhFT, but not GhPEBP2. The present analyses of protein structure, expression pattern and function support the conclusions that *GhFT* was flowering related promoter and *GhPEBP2* has no consistent effect on flowering.

In conclusion, this study highlights the important role of cotton *PEBP*-like genes in specifying photoperiod responsiveness and regulating the time of flowering. The results provide a method with which to distinguish cotton cultivars of different maturity classes through monitoring expression of important flowering-related genes. In addition, the findings provide insights into the mechanism by which the genes *GhFT* may regulate flowering time. Broadly, our results provide a better understanding for in-depth analyzing upland cotton breeding and guiding future work.

## Supporting Information

S1 Fig Genomic structures of *GhPEBP*-like genes. **(A)** Exons are indicated by black boxes and introns by a thin line. **(B)** Aligned amino acids of GhPEBPs in upland cotton.
(TIF)

S2 Fig Predicted structure of GhFT/GhTFL1s and GhPEBP proteins. The protein structure prediction was performed using SWISSMODEL.
(TIF)

S3 Fig Quantitative real-time PCR analysis of *Arabidopsis* flowering-related genes. Seedlings of wild-type and 35S::*GhFT* plants were grown for 14 days under long-day conditions.
(TIF)

S4 Fig Transcript levels of *GhPEBP2* in different transgenic lines. (TIF)

S5 Fig Promoter analysis of *GhFT* and *GhPEBP2*. Black boxes indicated exons, white boxes indicated introns. Gray boxes with numbers indicated promoter responsive elements.
(TIF)

S1 Table Accession numbers of all PEBP family members (multiple species). (XLSX)

S2 Table Primers used to amplify *GhPEBP*-like genes. (XLSX)
