# Supplementary figures and images for: Characterization and Functional Analysis of PEBP Family Genes in Upland Cotton (Gossypium hirsutum L.)
Source: PLoS One. 2016 Aug 23;11(8):e0161080. doi: 10.1371/journal.pone.0161080 (PMC4995033; doi:10.1371/journal.pone.0161080)

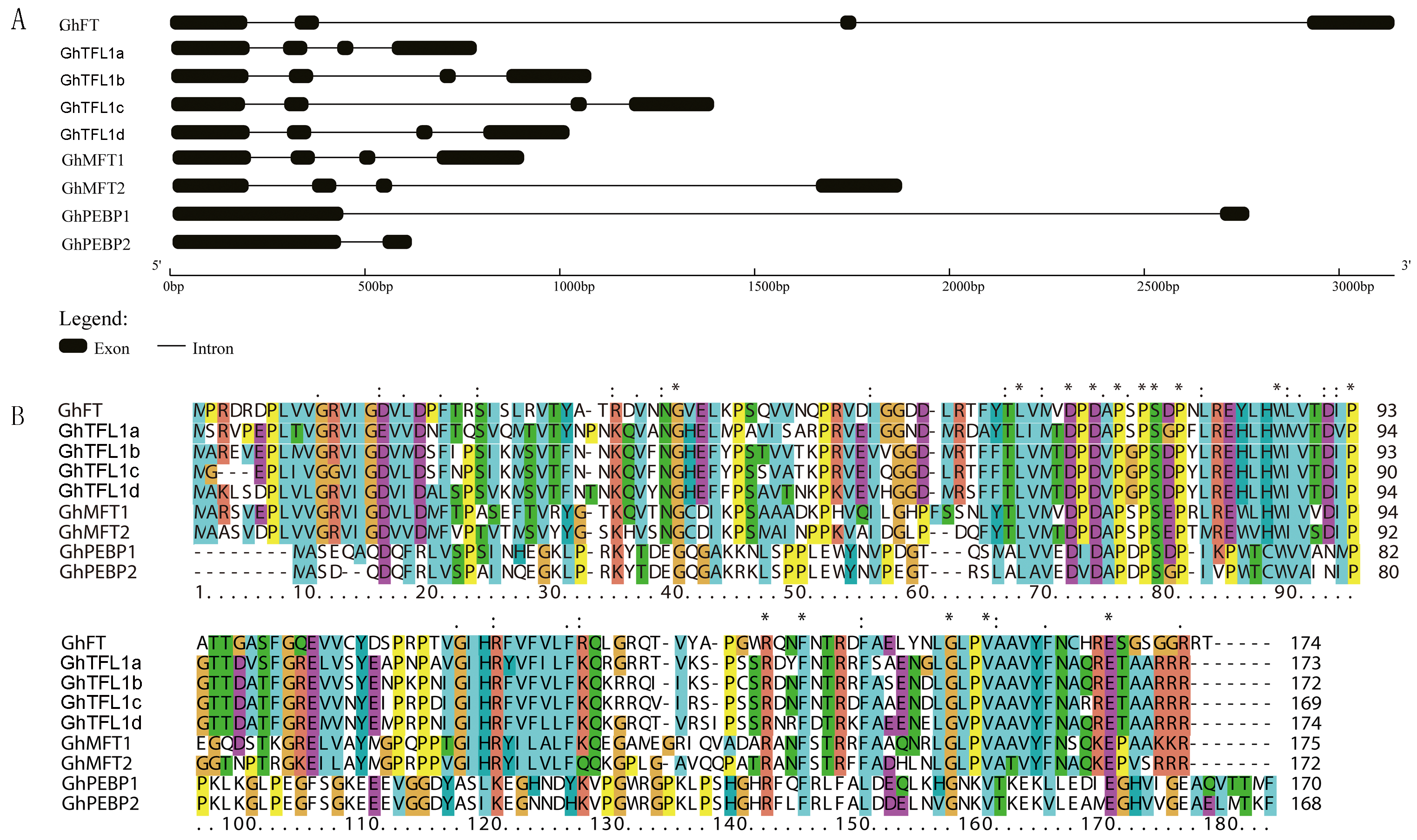

Supplement: S1 Fig — (A) Exons are indicated by black boxes and introns by a thin line. (B) Aligned amino acids of GhPEBPs in upland cotton. (TIF) [file pone.0161080.s001.tif]

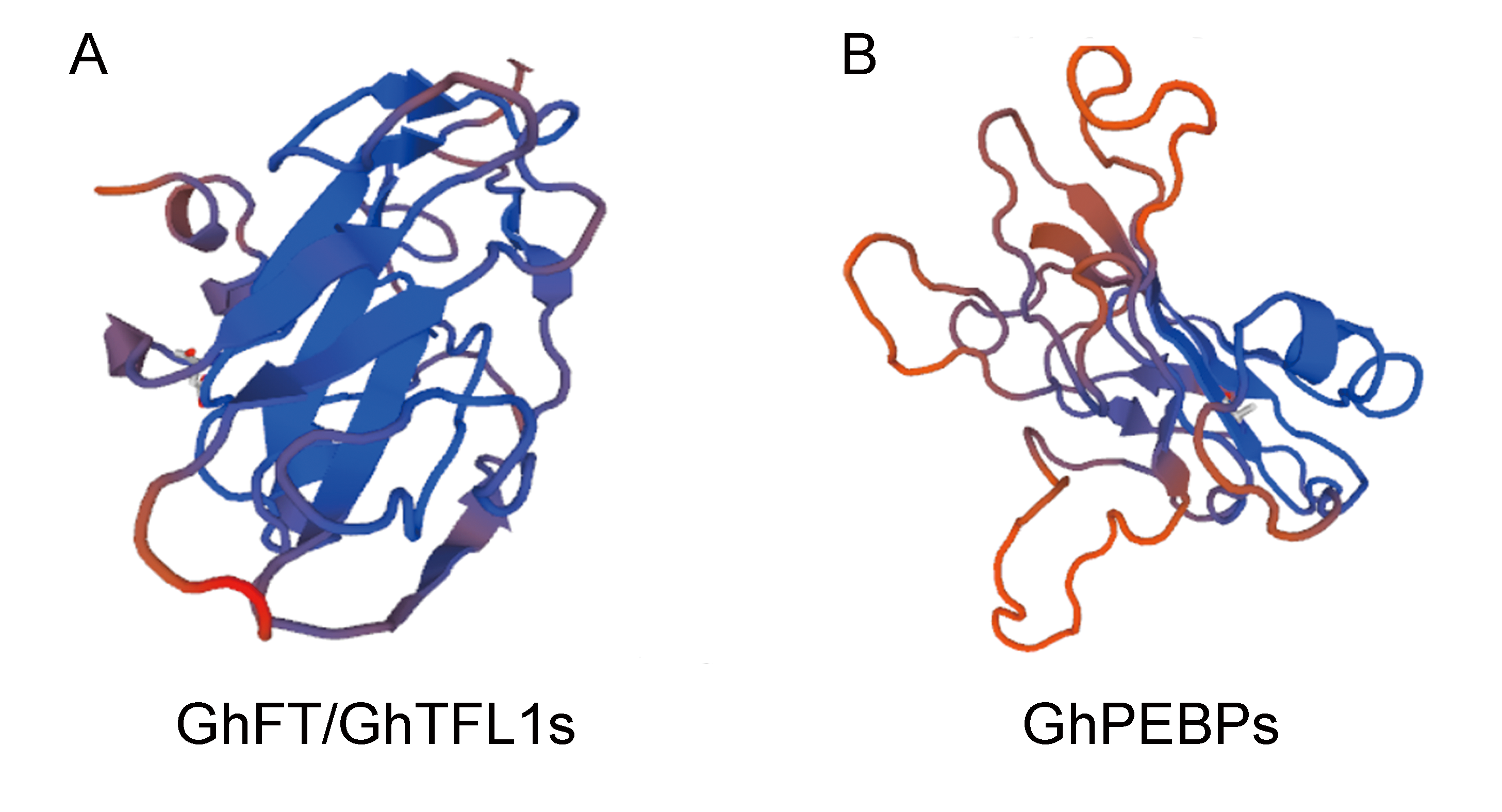

Supplement: S2 Fig — The protein structure prediction was performed using SWISSMODEL. (TIF) [file pone.0161080.s002.tif]

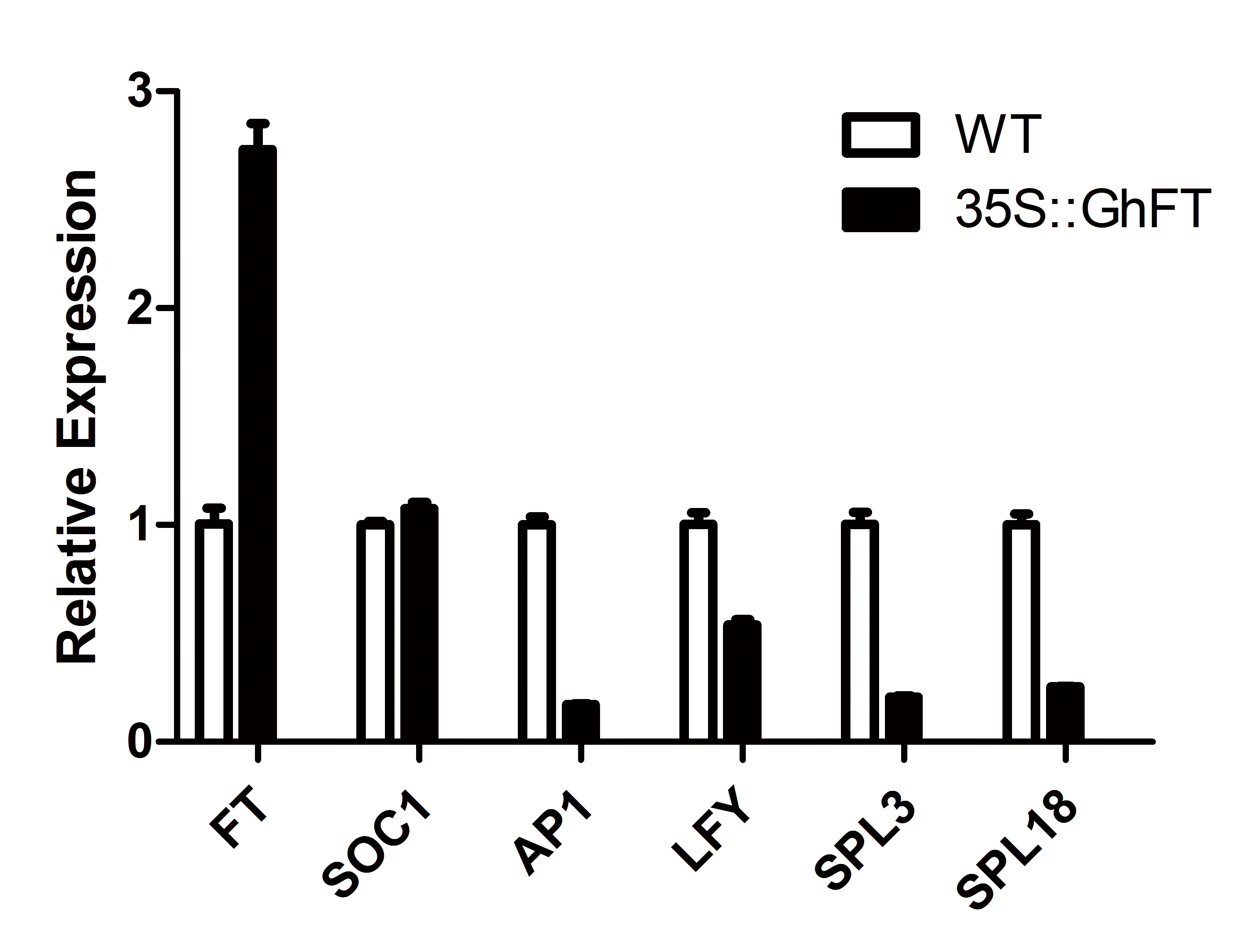

Supplement: S3 Fig — Seedlings of wild-type and 35S::GhFT plants were grown for 14 days under long-day conditions. (TIF) [file pone.0161080.s003.tif]

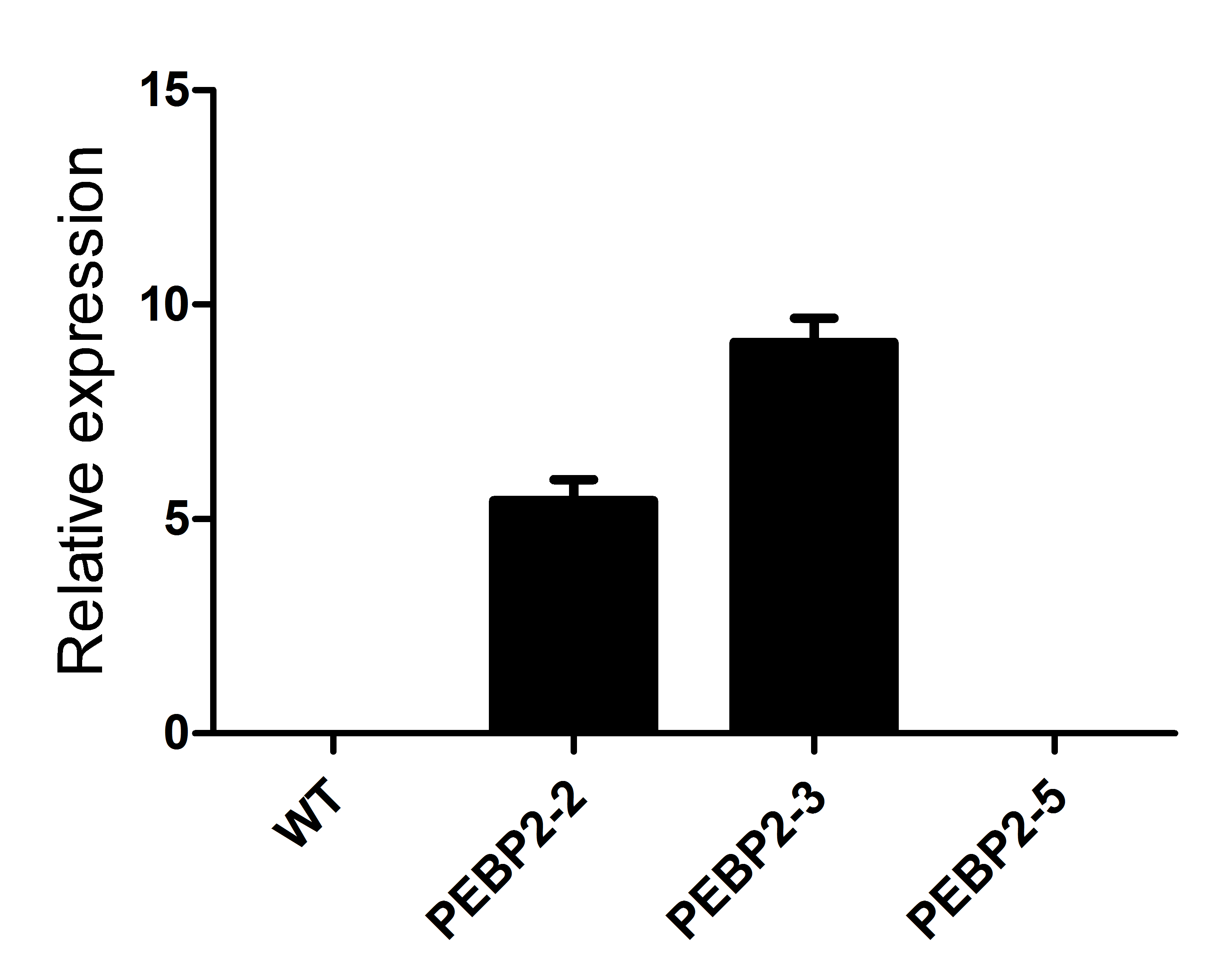

Supplement: S4 Fig — (TIF) [file pone.0161080.s004.tif]

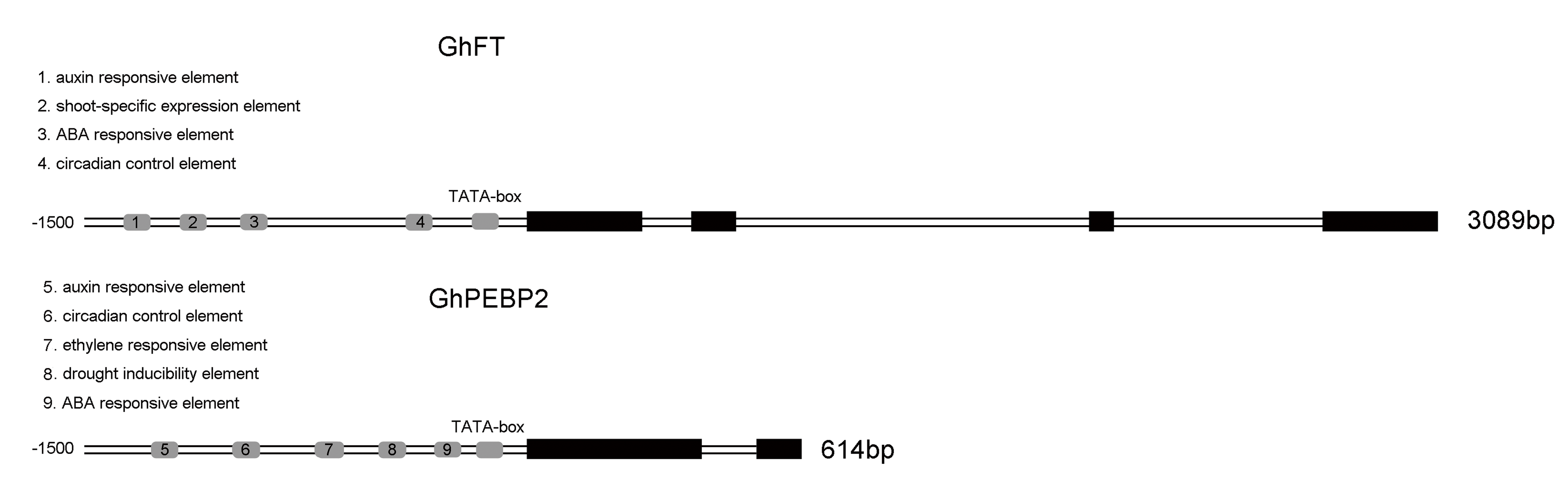

Supplement: S5 Fig — Black boxes indicated exons, white boxes indicated introns. Gray boxes with numbers indicated promoter responsive elements. (TIF) [file pone.0161080.s005.tif]
